# Research Progress and Clinical Practice in the Comorbidity Management of Obstructive Sleep Apnea Hypopnea Syndrome and Obesity Hypopnea Syndrome

**DOI:** 10.3390/diagnostics16030444

**Published:** 2026-02-01

**Authors:** Linlin Li, Ruixue Geng, Yuchen Wang, Jiafeng Wang

**Affiliations:** 1 Otolaryngology & Head and Neck Center, Cancer Center, Department of Head and Neck Surgery, Zhejiang Provincial People’s Hospital (Affiliated People’s Hospital), Hangzhou Medical College, Hangzhou 310014, China; 2 School of Clinical Medicine, Hangzhou Normal University, Hangzhou 311121, China; 3 School of Pharmacy, Hangzhou Medicine College, Hangzhou 310053, China; 4 Zhejiang Key Laboratory of Precision Medicine Research on Head & Neck Cancer, Hangzhou 310014, China; 5 Zhejiang Provincial Clinical Research Center for Head & Neck Cancer, Hangzhou 310014, China

**Keywords:** obstructive sleep apnea-hypopnea syndrome, obesity hypoventilation syndrome, comorbidity management, digital therapeutics, metabolic intervention, precision medicine

## Abstract

Obstructive Sleep Apnea-Hypopnea Syndrome (OSAHS) and Obesity Hypoventilation Syndrome (OHS) are core components of the obesity-related respiratory disease spectrum, and their comorbidity has become a major challenge in the global public health field. This review systematically summarizes the epidemiological characteristics, pathophysiological mechanisms, diagnostic criteria, diagnostic technologies and treatment strategies of OSAHS-OHS comorbidity, with a focus on the cutting-edge progress of digital therapeutics and metabolic intervention, as well as the historical evolution and current status of clinical management. We also conduct an in-depth analysis of the unresolved controversies and practical challenges in the current clinical management of this comorbidity. OSAHS-OHS comorbid patients have a significantly higher risk of cardiovascular complications than those with a single disease, and chronic intermittent hypoxia (CIH) forms a vicious cycle with obesity through multiple pathophysiological pathways. The combination of multi-dimensional assessment tools and portable monitoring devices has improved the screening efficiency of OSAHS-OHS comorbidity, and the selection of respiratory support therapies such as continuous positive airway pressure (CPAP) and non-invasive ventilation (NIV) depends on patient phenotypes. Digital therapeutics and novel metabolic intervention drugs have shown promising clinical value in the management of this comorbidity. The multidisciplinary collaboration model is the key to improving the prognosis of comorbid patients, while current clinical management is still faced with challenges such as policy lag, ethical controversies and uneven resource allocation. Future research should focus on individualized therapeutic targets, the integration of digital technologies and the optimization of health policies to achieve precise and efficient management of OSAHS-OHS comorbidity.

## 1. Introduction

With the continuous rise in the global prevalence of obesity, the comorbidity of Obstructive Sleep Apnea-Hypopnea Syndrome (OSAHS) and Obesity Hypoventilation Syndrome (OHS) has become a major public health threat, with a significantly higher risk of cardiovascular and metabolic complications than either single disease. Specifically, clinical studies have confirmed that the all-cause mortality rate of OSAHS+OHS patients after percutaneous coronary intervention (PCI) is 2.8%, which is significantly higher than 1.1% in patients with OSAHS alone [1], underscoring the severe clinical sequelae of this comorbidity.

At the pathophysiological level, chronic intermittent hypoxia (CIH)—a hallmark feature of OSAHS—forms a detrimental vicious cycle with obesity in OSAHS+OHS comorbidity via oxidative stress, excessive inflammatory response, and aberrant metabolic reprogramming. CIH can accelerate adipogenesis and adipose tissue dysfunction, while obesity further exacerbates upper airway obstruction and hypoventilation, thereby amplifying the pathological progression of both syndromes [2]. This pathological interplay not only explains the elevated complication risk but also highlights the complexity of comorbidity management.

In clinical practice, advances in diagnostic and therapeutic technologies have provided new tools for OSAHS+OHS management. The combination of multi-dimensional assessment tools (e.g., the STOP-Bang questionnaire and Epworth Sleepiness Scale) and portable monitoring devices has improved screening efficiency, yet serum bicarbonate level (≥27 mmol/L)—a common OHS predictive indicator—still has gender differences in diagnostic efficacy, limiting its universal application [3]. For treatment, the selection of continuous positive airway pressure (CPAP) and non-invasive ventilation (NIV) depends on patient phenotypes, and digital therapeutics (e.g., remote real-time monitoring) can increase long-term CPAP adherence to 80.66% [4]. Meanwhile, metabolic interventions show promising potential: sodium-glucose cotransporter 2 inhibitors (SGLT2i) reduce endogenous CO_2_ production to improve metabolic disorders [5], and glucagon-like peptide-1 receptor agonists (GLP-1RA) optimize airway structure through weight loss [6].

Despite these advances, traditional comorbidity management still faces many challenges, including diagnostic delay, poor treatment adherence, and single intervention goals, which highlights the urgent need to establish a more efficient management framework. The core viewpoint of this review is "precision management driven by the integration of digital and metabolic approaches"—that is, using digital technologies (such as remote monitoring and artificial intelligence-based phenotypic analysis) to accurately classify and real-time assess comorbid patients, and combining metabolic interventions (such as new drugs and weight loss strategies) to target the core pathological links of the disease, ultimately breaking the vicious cycle of "hypoxia-inflammation-metabolic dysfunction".

Subsequent chapters will be carried out around this core framework, sequentially discussing epidemiological characteristics, pathophysiological mechanisms, diagnostic criteria, diagnostic technologies, treatment strategies, and controversial issues in clinical management, focusing on the cutting-edge progress of digital therapeutics and metabolic intervention. This structure ensures that all content is highly consistent with the core viewpoint, and aims to provide systematic practical and research ideas for clinicians and researchers.

## 2. Methods for Literature Review

### 2.1. Search Terms

Core terms: obstructive sleep apnea hypopnea syndrome, obesity hypoventilation syndrome, OSAHS, OHS; comorbidity and management-related terms: comorbidity, coexistence, combined syndrome, management, treatment, CPAP, non-invasive ventilation, weight loss intervention; Boolean combination strategy: (OSAHS OR obstructive sleep apnea) AND (OHS OR obesity hypoventilation) AND (comorbidity OR coexistence OR combined), supplemented by: (CPAP OR NIV OR bPAP) AND (management OR outcome OR guideline). All terms were applied with truncation (*) and proximity operators to capture variant forms (e.g., “treat*”, “manag*”).

### 2.2. Databases

PubMed, Embase, Cochrane Library, Web of Science, Scopus—all databases are indexed in JCR and endorsed by the American Academy of Sleep Medicine (AASM) and European Respiratory Society (ERS) for systematic review conduct.

### 2.3. Selection Process

Inclusion criteria: Studies explicitly addressing OSAHS, OHS, or their coexistence in adult populations (≥18 years); focus on diagnostic criteria, therapeutic interventions, or long-term management outcomes. Exclusion criteria: Animal models, in vitro studies.

### 2.4. Scope

Time frame: 2009–2025. Priority was given to randomized controlled trials, cohort studies, systematic reviews, and meta-analyses, with consideration of innovative proof-of-concept studies.

## 3. Basic Theory

### 3.1. Epidemiological Characteristics

The comorbidity of OSAHS and OHS is particularly prominent in obese populations, and its epidemiological characteristics show significant population heterogeneity and disease correlation. A retrospective study involving 989 patients with sleep-disordered breathing showed that the prevalences of OSAHS, OSAHS-Chronic Obstructive Pulmonary Disease (COPD) overlap syndrome, and OSAHS + OHS were 72.9%, 12.4%, and 14.7%, respectively. Among them, patients with OSAHS + OHS had the highest incidence of multiple comorbidities (≥2 types) (68.3%), and the prevalences of metabolic diseases such as arterial hypertension (89.7%) and diabetes mellitus (42.1%) were significantly higher than those in patients with OSAHS alone [7]. Further analysis found that although the average age of patients with OSAHS + OHS (48.2 ± 11.5 years) was lower than that of patients with OSAHS alone (52.6 ± 10.8 years), their BMI (41.3 ± 6.2 kg/m^2^) was significantly higher, and excessive daytime sleepiness (Epworth Sleepiness Scale score ≥ 10 points) was positively correlated with the risk of multiple comorbidities (OR = 1.89, 95%CI: 1.23–2.91) [7]. In the pediatric population, the prevalence of OSAHS is 12.8%, among which the Apnea-Hypopnea Index (AHI) of patients with Positional OSA (POSA) is significantly lower than that of patients with non-positional OSAHS (8.66 vs. 10.30, *p* < 0.05), and obesity (BMI z-score ≥ 2) is an independent risk factor for POSA (OR = 2.34, 95%CI: 1.56–3.51) [8]. It is worth noting that the comorbidity of OSAHS and OHS is also closely related to the disease burden of specific populations. For example, the incidence of OSAHS in patients with acromegaly is as high as 52.0%, and the thickness of the posterior pharyngeal wall is positively correlated with the level of insulin-like growth factor-1 (IGF-1) (soft palate plane slope = 0.001, *p* = 0.006) [9]; while in patients with primary open-angle glaucoma (POAG), the prevalence of OSAHS is 49%, suggesting that sleep-disordered breathing may be involved in optic nerve damage through vascular mechanisms [10].

The comorbidity of OSAHS and OHS is not only characterized by high prevalence, but also by the synergistic effect of disease severity and complication risk. A study based on the HypnoLaus cohort showed that the proportion of Positional OSA (POSA) among OSAHS patients is as high as 75%. Among them, postmenopausal status in women is associated with a reduced risk of POSA (OR = 0.62, 95%CI: 0.41–0.94), while for each 1 kg/m^2^ increase in BMI in men, the risk of POSA increases by 1.16 times [11]. In patients with OSAHS + OHS, the incidence of nocturnal hypoxemia (minimum blood oxygen saturation < 80%) is 82.8%, which is significantly higher than 54.5% in patients with OSAHS alone, and the diagnostic sensitivity and specificity of serum bicarbonate level ≥27 mmol/L for OHS are 76.6% and 74.6%, respectively [12]. In terms of cardiovascular complications, the incidence of cardiogenic shock after PCI in patients with OSAHS + OHS is 7.3%, which is significantly higher than that in patients with OSAHS-COPD overlap syndrome (3.4%) and OSAHS alone (2.6%) [1]; while in OSAHS patients with heart failure with preserved ejection fraction (HFpEF) and atrial fibrillation (AF), the right atrial diameter (RAA) is positively correlated with the severity of OSAHS (r = 0.42, *p* < 0.01) [13]. At the metabolic disorder level, the intestinal flora structure of OSAHS patients undergoes significant changes. For example, the ratio of Firmicutes/Bacteroidetes increases (SMD = 0.58, 95%CI: 0.32–0.84), and the level of fecal metabolites such as Formononetin is positively correlated with AHI (AUC = 0.9100, sensitivity 82.5%, specificity 90.0%) [14,15]. In addition, the Fear of Progression (FOP) score of OSAHS patients is negatively correlated with health-promoting behaviors (r = −0.55, *p* < 0.001), and social support can alleviate this association through a mediating effect (accounting for 59.00%) [16].

### 3.2. Pathophysiological Mechanisms

The pathophysiological mechanisms of OSAHS and OHS comorbidity involve complex interactions of mechanical airway obstruction, neuroendocrine disorders, and systemic inflammation. In terms of mechanical factors, obesity-induced reduction in chest wall compliance (decreased by 20–30%) and functional residual capacity (decreased by 15–20%) significantly lower the airway collapse threshold [17]. At the same time, the deposition of adipose tissue in the pharynx (such as a 1.2 mm increase in the thickness of the posterior pharyngeal wall) directly reduces the cross-sectional area of the airway, while CIH promotes fibroblast proliferation by activating hypoxia-inducible factor-1α (HIF-1α), further aggravating airway remodeling [9,18]. At the neuroendocrine level, leptin resistance is the core mechanism of weakened central respiratory drive in OHS patients. Studies have shown that the sleep hypoventilation index of db/db mice (leptin receptor deficiency) is significantly increased (AHI = 58.2 vs. 12.1 in the control group, *p* < 0.001), and restoring leptin signaling in the dorsomedial hypothalamus (DMH) region can increase minute ventilation by 25% [19,20]. In addition, chronic hypercapnia accelerates adipogenesis through the soluble adenylyl cyclase (sAC)-cAMP pathway. For example, the expression of the adipogenic marker PPARγ in human visceral preadipocytes increases by 2.3 times under 5% CO_2_ environment, forming a vicious cycle of “obesity-hypercapnia-obesity” [2].

**Figure 1:**
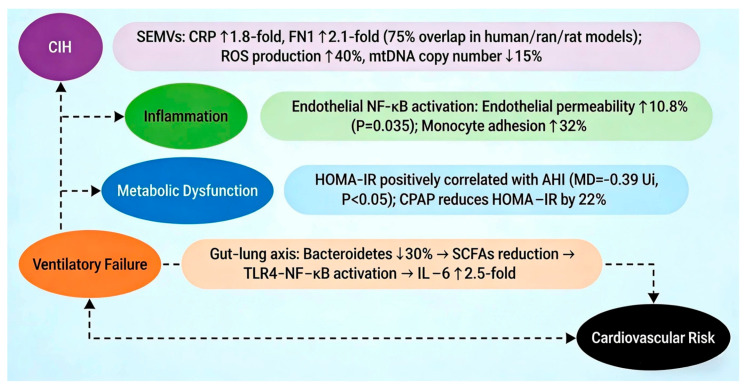
Integrated model of CIH-mediated disease progression in OSAHS-OHS comorbidity. Chronic intermittent hypoxia (CIH) serves as the initiating factor, activating oxidative stress and inflammatory pathways (e.g., increased CRP levels and NF-κB pathway activation). This triggers metabolic dysfunction, including insulin resistance and enhanced adipogenesis. Subsequently, combined with leptin resistance and airway structural damage, ventilatory failure develops. Finally, long-term exposure to hypoxia, inflammation, and metabolic abnormalities leads to endothelial dysfunction, left ventricular hypertrophy, arrhythmia, and other cardiovascular complications, forming a self-reinforcing vicious cycle. This integrated model clearly reveals the core logic of comorbidity from initiation to progression, with each link promoting and amplifying each other, providing clear targets for targeted intervention [15,18,21,22,23,24].

Systemic inflammation and oxidative stress play key roles in the progression of OSAHS and OHS comorbidity. CIH can induce abnormal expression of inflammatory proteins in serum extracellular microvesicles (SEMVs). For example, the levels of C-reactive protein (CRP) and fibronectin (FN1) in SEMVs of OSAHS patients are increased by 1.8 times and 2.1 times, respectively, and the change trends of the same proteins in CIH rat models are consistent (overlap rate up to 75%) [21]. These SEMVs cause vascular endothelial dysfunction by activating the endothelial cell NF-κB pathway, manifested as a 10.8% increase in endothelial permeability (*p* = 0.035) and a 32% increase in monocyte adhesion rate [22]. In terms of oxidative stress, the production rate of reactive oxygen species (ROS) in OSAHS patients is 40% higher than that in the control group, and ROS further inhibits respiratory chain function by damaging mitochondrial DNA (mtDNA) with a 15% reduction in copy number [18,23]. At the metabolic disorder level, the Homeostasis Model Assessment of Insulin Resistance (HOMA-IR) of OSAHS patients is positively correlated with AHI (MD = −0.39 Ui, *p* < 0.05), and CPAP treatment can reduce HOMA-IR by 22% by improving sleep structure [24]. In addition, intestinal flora imbalance exacerbates inflammatory response through the “gut–lung axis”. For example, the abundance of Bacteroidetes in the feces of OSAHS patients decreases by 30%, and the reduction of its metabolite short-chain fatty acids (SCFAs) can lead to the activation of the TLR4-NF-κB pathway, resulting in a 2.5-fold increase in the level of the pro-inflammatory factor IL-6 [15] (Figure 1).

### 3.3. Diagnostic Criteria

**Figure 2:**
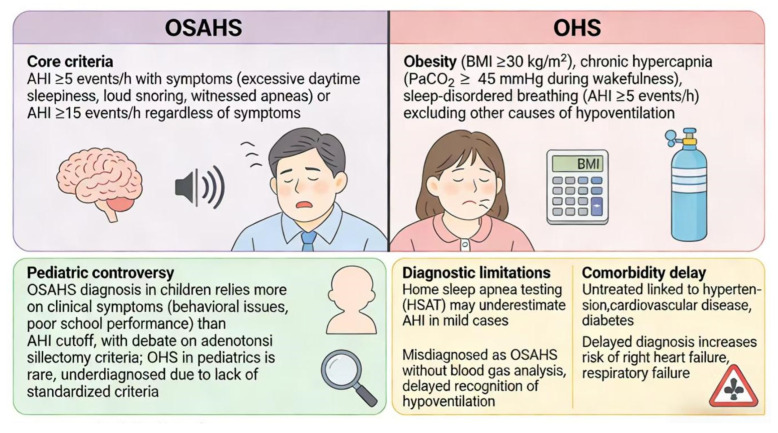
Visual summary of OSAHS and OHS diagnostic pathways and pitfalls. This infographic contrasts the diagnostic criteria for obstructive sleep apnea-hypopnea syndrome (OSAHS) and obesity hypoventilation syndrome (OHS), emphasizing the role of AHI, BMI, and blood gas analysis. It addresses pediatric diagnostic controversies, including the reliance on clinical symptoms over AHI cutoffs in children, and highlights limitations of home sleep apnea testing (HSAT) in detecting mild OSAHS. The infographic also underscores the high burden of unrecognized OSAHS-OHS comorbidity, with 42.1% of obese OSAHS patients having undiagnosed OHS [12]. All clinical data are supported by references [3,8,12,25,26].

The diagnosis of OSAHS and OHS needs to combine clinical symptoms, sleep monitoring, and blood gas analysis, but current standards still face many challenges. The core diagnostic criterion for OSAHS is AHI ≥ 5 events/hour, accompanied by daytime sleepiness (Epworth score ≥ 10) or related complications [25]. According to the guidelines of the American Academy of Sleep Medicine (AASM), AHI ≥ 15 events/hour may be sufficient for the diagnosis of OSAHS even in the absence of symptoms [25]. However, the diagnostic threshold for pediatric OSAHS is controversial. For example, a study involving 1236 children showed that POSA accounts for 12.8% of patients with AHI ≥ 1 event/hour, but there is still no consensus on whether patients with non-supine AHI < 5 events/hour need treatment [8]. The diagnostic criteria for OHS are BMI ≥ 30 kg/m^2^, daytime PaCO_2_ ≥ 45 mmHg, and exclusion of hypoventilation from other causes. However, its differentiation from OSAHS relies on blood gas analysis, and serum bicarbonate level as a surrogate indicator has gender differences—the average bicarbonate level in men (25.2 mmol/L) is significantly higher than that in women (24.4 mmol/L), and BMI is negatively correlated with bicarbonate (decreased by 0.03 mmol/L for each 1 kg/m^2^ increase, *p* < 0.001) [3,26]. In addition, the comorbid diagnosis of OSAHS and OHS is often delayed due to overlapping symptoms. For example, a study showed that 42.1% of obese OSAHS patients have comorbid OHS, but only 28% were diagnosed during the first sleep monitoring [12] (Figure 2).

The limitations of diagnostic technologies further exacerbate the difficulty in identifying OSAHS and OHS comorbidity. Although traditional polysomnography (PSG) is the gold standard, it is not easily accessible in primary medical institutions, and the diagnostic accuracy of home sleep apnea testing (HSAT) is limited by monitoring parameters (such as only recording respiratory events without electroencephalography) [27]. For example, a study involving 157 patients with suspected OSAHS showed that the median diagnostic delay of HSAT was 11.7 days, and 36% of patients failed to complete the test due to difficulty in equipment operation [28]. In terms of biomarkers, miRNA profile analysis showed that the serum levels of miR-126-3p and miR-26a-5p in OSAHS patients with hypertension were significantly increased (AUC 0.85 and 0.82, respectively), but the specificity of these biomarkers still needs to be verified [29]. In addition, patients with comorbid OSAHS and OHS are often accompanied by multiple comorbidities. For example, 49% of POAG patients have comorbid OSAHS, but the screening rate of ophthalmologists for sleep symptoms is only 18% [10]; while in patients with mental illnesses, the screening rate of OSAHS is less than 30%, and even after diagnosis, the CPAP adherence rate is only 42% [30]. For OSAHS patients with severe obesity (body mass index [BMI] ≥ 40 kg/m^2^), persistent daytime sleepiness after OSAHS treatment, unexplained fatigue, or serum bicarbonate level ≥ 27 mmol/L, Obesity Hypoventilation Syndrome (OHS) should be suspected [12,31]. Such patients should undergo arterial blood gas analysis, especially when polysomnography or polyphysiological monitoring results show severe nocturnal hypoxemia (minimum oxygen saturation < 80%) or prolonged hypercapnia (transcutaneous carbon dioxide partial pressure >50 mmHg, duration exceeding 10% of sleep time) [27,31].

## 4. Clinical Practice

### 4.1. Comprehensive Diagnostic Technologies

The comprehensive diagnostic technology system for OSAHS and OHS includes screening tools, sleep monitoring, and blood gas analysis, aiming to achieve early identification and precise typing. At the screening level, the combination of the STOP-Bang questionnaire (sensitivity 83%, specificity 74%) and the Epworth Sleepiness Scale (ESS) can effectively identify high-risk populations. For example, a study involving 129 obese hypoxemic patients showed that the prevalence of OSAHS + OHS in patients with a STOP-Bang score ≥5 was 68% [32,33]. In terms of sleep monitoring, the application of portable devices has significantly improved diagnostic efficiency. For example, a machine learning model based on snoring sounds (VGG16 + PANN fusion) has a diagnostic accuracy of 100% for OSAHS, and the average prediction time of the Gaussian mixture model is only 0.134 ± 0.005 s [34,35]. Polysomnography (PM) can record respiratory events, blood oxygen saturation, and carbon dioxide levels simultaneously. For example, the correlation between transcutaneous CO_2_ monitoring (PtcCO_2_) and arterial blood gas is 0.86 (*p* < 0.001), and the duration of nocturnal PtcCO_2_ > 50 mmHg is positively correlated with daytime PaCO_2_ (r = 0.72, *p* < 0.001) [27,36].

Innovations in diagnostic technologies have provided new dimensions for the comorbid typing of OSAHS and OHS. For example, CT-based three-dimensional reconstruction of the upper airway shows that the soft palate volume of patients with OSAHS + OHS (28.6 ± 5.2 cm^3^) is significantly larger than that of patients with OSAHS alone (21.3 ± 4.7 cm^3^, *p* < 0.001), and the minimum cross-sectional area of the airway is negatively correlated with BMI (r = −0.61, *p* < 0.001) [9]. Metabolomic analysis found that there are differences in 1164 metabolites in the feces of 4–6-year-old OSAHS children, among which the level of Formononetin is positively correlated with OAHI (r = 0.78, *p* < 0.001), which can be used as an early diagnostic biomarker [14]. In addition, remote monitoring technology in digital therapeutics can real-time evaluate treatment response. For example, a study involving 579 OSAHS patients showed that the CPAP adherence rate under remote monitoring was 80.66%, and there was no significant difference in adherence rate between elderly patients (>65 years old) and young patients (6.6 vs. 6.7 h/night, *p* = 0.89) [4]. However, the application of diagnostic technologies still faces challenges. For example, as a predictive indicator for OHS, the specificity of serum bicarbonate level is only 68% in women and 78% in men [3]; and the diagnostic accuracy of HSAT is reduced by 25% in patients with comorbid severe lung diseases [27].

### 4.2. Traditional Treatment Strategies

**Figure 3:**
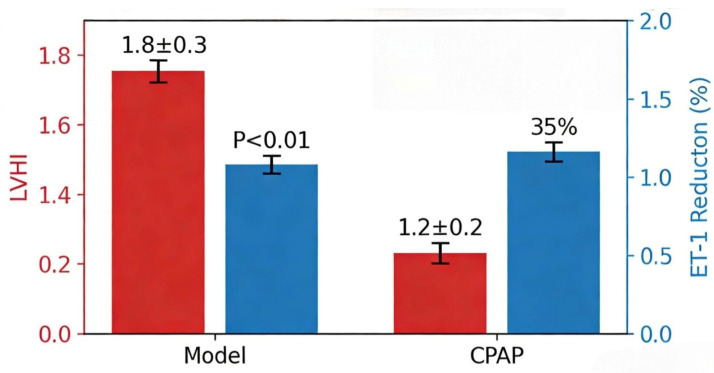
CPAP Treatment Attenuates Left Ventricular Hypertrophy and Reduces Plasma Endothelin-1 Level in a Rabbit Model of OSAHS This bar graph compares the left ventricular hypertrophy index (LVHI) and relative reduction rate of plasma endothelin-1 (ET-1) between OSAHS model rabbits and those receiving continuous positive airway pressure (CPAP) treatment (*n* = 30 New Zealand rabbits). The OSAHS model group exhibited an LVHI of 1.8 ± 0.3, while CPAP treatment significantly reduced LVHI to 1.2 ± 0.2 (*p* < 0.01). Additionally, CPAP treatment decreased plasma ET-1 levels by 35% compared with the model group. Error bars represent standard deviation (SD). Color explanation: Red bars represent the left ventricular hypertrophy index (LVHI), and blue bars represent the relative reduction rate of plasma endothelin-1 (ET-1).

The traditional treatment strategies for OSAHS and OHS are centered on positive airway pressure ventilation, supplemented by lifestyle interventions and surgical treatment. As the first-line treatment for OSAHS, CPAP can significantly reduce the risk of cardiovascular complications. For example, a study involving 30 New Zealand rabbits showed that the left ventricular hypertrophy index (LVHI) of the OSAHS model group was 1.8 ± 0.3, while that of the CPAP treatment group decreased to 1.2 ± 0.2 (*p* < 0.01) (Figure 3), and the plasma endothelin-1 (ET-1) level decreased by 35% [37]. For OHS patients, the application of NIV can improve daytime PaCO_2_. A randomized controlled trial showed that after 2 months of NIV treatment, the PaCO_2_ of patients decreased by 6 mmHg (95%CI: −7.7 to −4.2, *p* < 0.001) and the sleep quality score (SF-36) increased by 18 points [38]. In terms of lifestyle interventions, surgical treatment for comorbid OSAHS and OHS should focus on obesity-related pathological mechanisms, among which bariatric surgery plays a key role. Bariatric surgery (such as sleeve gastrectomy and Roux-en-Y gastric bypass) fundamentally reverses obesity by reducing gastric capacity and changing nutrient absorption, thereby improving chest wall compliance, reducing pharyngeal fat deposition, and alleviating airway obstruction [39]. Weight loss is a basic measure. For example, metabolic bariatric surgery can reduce the AHI of OSAHS patients by 45% (from 51.1 to 28.1, *p* < 0.001), improve insulin resistance, reduce the level of serum inflammatory factors, break the cycle of “obesity-hypoxia-metabolic dysfunction”, and the 3-year remission rate after surgery is 68% [39]. In addition, positional therapy is effective for POSA patients. For example, the use of positional correction devices can increase non-supine sleep time by 40 min and reduce AHI by 31% [11].

**Figure 4:**
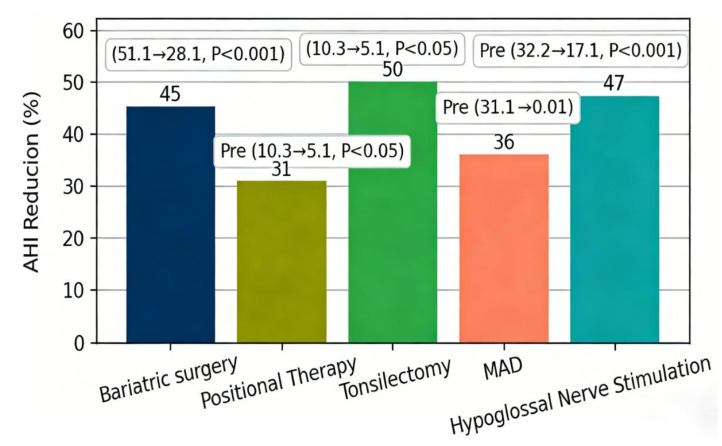
Comparison of Apnea-Hypopnea Index (AHI) Reduction Rates Among Lifestyle Interventions and Surgical Treatments for Patients with Obstructive Sleep Apnea-Hypopnea Syndrome (OSAHS) This bar chart illustrates the percentage reduction in apnea-hypopnea index (AHI) following different lifestyle and surgical interventions for OSAHS patients. Bariatric Surgery: Reduced AHI by 45% (from 51.1 to 28.1, *p* < 0.001), with a 68% 3-year remission rate for OSAHS. Positional Therapy: Decreased AHI by 31% in positional OSA (POSA) patients, while increasing non-supine sleep duration by 40 min. Tonsillectomy: Achieved a 50% AHI reduction (from 10.3 to 5.1, *p* < 0.05) in pediatric OSAHS patients, and improved height Z-score by 0.3. Mandibular Advancement Device (MAD): Reduced AHI by 36% and decreased myocardial endothelin-1 (ET-1) mRNA expression by 2.1-fold. Hypoglossal Nerve Stimulation: Reduced AHI by 47% (from 32.2 to 17.1, *p* < 0.001) in patients with refractory OSAHS, though its efficacy in obesity hypoventilation syndrome (OHS) patients remains to be validated.

Surgical treatment has important value in specific patient populations. For example, tonsillectomy can reduce the AHI of pediatric OSAHS patients by 50% (from 10.3 to 5.1, *p* < 0.05) and increase growth and development indicators (such as height z-score) by 0.3 [40]. In terms of upper airway surgery, the Mandibular Advancement Device (MAD) can reduce the AHI of OSAHS patients by 36% and decrease the myocardial ET-1 mRNA expression by 2.1 times [37]. For patients with refractory OSAHS, hypoglossal nerve stimulation can reduce AHI by 47% (from 32.2 to 17.1, *p* < 0.001) (Figure 4), but the application of this technology in OHS patients still needs to be verified [41]. Compared with other surgical methods (such as tonsillectomy and mandibular advancement device), bariatric surgery has a more durable effect on comorbid patients, especially for patients with severe obesity (body mass index [BMI] ≥ 40 kg/m^2^) [37,40]. However, bariatric surgery is not suitable for all comorbid patients; contraindications include severe cardiopulmonary dysfunction, coagulation disorders, and mental illnesses. For patients who are not suitable for bariatric surgery, minimally invasive upper airway surgery (such as uvulopalatopharyngoplasty) can be considered as adjuvant treatment, but its efficacy is limited by the degree of obesity, and long-term weight management is still needed to maintain the surgical effect [41]. However, traditional treatment strategies still have limitations. For example, the CPAP adherence rate in OHS patients is only 56%, which is significantly lower than 72% in patients with OSAHS alone [4]; while the cost of NIV treatment is high (annual average $147,209), which limits its application in low-income areas [1]. In addition, although drug treatments such as SGLT2i can reduce endogenous CO_2_ production, clinical trials in OHS patients are still in the early stage [5].

### 4.3. Controversial Issues in Clinical Management

The clinical management controversies of OSAHS and OHS mainly focus on three aspects: treatment plan selection, adherence improvement, and comorbidity management. In terms of treatment plan selection, there is still disagreement on the target populations of CPAP and NIV: a randomized controlled trial involving 86 OHS patients showed that there was no significant difference between CPAP and NIV in improving PaCO_2_ (decreased by 6 mmHg and 5.8 mmHg, respectively, *p* = 0.89) and quality of life, but the equipment cost of the NIV group was higher (annual average $10,200 vs. $6800 in the CPAP group) [38,42]. In terms of adherence, although remote monitoring technology can increase the CPAP adherence rate to 80.66%, the mask leakage rate of elderly patients (>65 years old) is significantly higher than that of young patients (45.7% vs. 24.9%, *p* < 0.01) [4]. In addition, drug treatment for patients with OSAHS + OHS has risks. For example, low-dose morphine (0.035 mg/kg) can cause respiratory depression, and multimodal analgesia (such as non-steroidal anti-inflammatory drugs + nerve block) should be used [43].

Controversies in comorbidity management are mainly reflected in the management of cardiovascular and metabolic diseases. For example, the mortality rate of patients with OSAHS + OHS after PCI is significantly higher than that of patients with OSAHS alone (2.8% vs. 1.1%, *p* < 0.001), but there is currently no optimized antiplatelet treatment plan for this population [1]. In terms of metabolic intervention, although GLP-1RA can reduce the BMI of OSAHS patients by 5.2 kg/m^2^ and AHI by 32%, long-term safety (such as pancreatitis risk) still needs to be observed [6]. In addition, there is controversy over the oxygen therapy strategy for OHS patients. A study showed that supplementary oxygen can increase PaO_2_ by 10 mmHg, but at the same time cause PaCO_2_ to increase by 5 mmHg, so the oxygen concentration must be strictly controlled (FiO_2_ < 30%) [44]. At the ethical level, the driving safety of patients with OSAHS+OHS has attracted much attention. Studies have shown that the traffic accident rate of this population is 46.5%, which is significantly higher than 26.9% of OSAHS patients (*p* = 0.029), but currently only 12% of countries include OSAHS in driving license screening [45].

### 4.4. Evidence Grading and Treatment Differentiation

To avoid overinterpretation in clinical practice, it is necessary to clarify the evidence levels of different intervention methods:

Conclusive clinical evidence: The airway patency effect of continuous positive airway pressure (CPAP) on OSAHS, the correction effect of non-invasive ventilation (NIV) on severe hypercapnia, and the long-term efficacy of bariatric surgery in patients with comorbid severe obesity have all been supported by sufficient randomized controlled trials and cohort studies, thus becoming the preferred clinical options [1,38,39].

Emerging but unproven therapies: Glucagon-like peptide-1 receptor agonists (GLP-1RA) and sodium-glucose cotransporter 2 inhibitors (SGLT2i) have shown potential value in comorbidity management, but their long-term safety (such as pancreatitis risk) and accurate classification of applicable populations still need to be verified in large-sample, long-term follow-up studies; currently, they are only recommended for some patients with comorbid metabolic diseases [5,6].

Translational or experimental methods: Leptin receptor agonists, hypoglossal nerve stimulation, and virtual reality-assisted treatment are still in the proof-of-concept or small-sample trial stage, and clinical application standards have not been formed; they are only suitable for refractory cases or clinical research scenarios [20,28,41].

## 5. Application of Digital Therapeutics

### 5.1. Current Status of Digital Therapeutics in Management

The application of digital therapeutics in the management of OSAHS and OHS has covered the entire process of screening, diagnosis, treatment, and follow-up. At the screening level, machine learning-based snoring sound analysis models can achieve preliminary home diagnosis. For example, the model integrating VGG16 and PANN features has a diagnostic accuracy of 100% for OSAHS, and the average prediction time of the Gaussian mixture model is only 0.134 ± 0.005 s [34,35]. In terms of diagnosis, remote sleep monitoring devices (such as mattresses with sensors) can transmit data to the cloud in real time. For example, a study involving 157 patients showed that the median time from the first remote consultation to HSAT diagnosis was 18.9 days, which was significantly shorter than the 32 days of the traditional process [28]. In terms of treatment, the remote monitoring function of CPAP devices can adjust pressure parameters in real time. For example, a multicenter study showed that the CPAP adherence rate under remote monitoring was 80.66%, and there was no significant difference in adherence rate between elderly patients (>65 years old) and young patients (6.6 vs. 6.7 h/night, *p* = 0.89) [4]. In addition, mobile applications in digital therapeutics (such as the HERB system) can reduce blood pressure through lifestyle interventions, indirectly improving the cardiovascular risk of OSAHS patients. For example, the system can reduce 24 h ambulatory systolic blood pressure by 2.4 mmHg (95%CI: −4.5 to −0.3, *p* < 0.05) [46].

The application of digital therapeutics still faces several challenges. For example, the accuracy of remote monitoring devices is limited by network stability. A study involving 12 patients with OSAHS + OHS showed that when the network delay >500 ms, the error rate of device data increased by 15% [22]. In terms of patient acceptance, a survey showed that 36% of elderly patients refused to use remote monitoring devices due to technical operation difficulties [4]. In addition, the cost-effectiveness of digital therapeutics still needs to be verified. For example, a randomized controlled trial showed that the annual medical cost of the remote monitoring group increased by $2300, but the hospitalization rate decreased by 35% (IRR = 0.55, 95%CI: 0.33–0.93) [33]. At the ethical level, the privacy protection of patient data is a key issue. For example, the Health Insurance Portability and Accountability Act (HIPAA) in the United States requires digital therapeutic platforms to have end-to-end encryption functions, but currently only 68% of platforms meet the standards [28].

### 5.2. Efficacy Evaluation of Digital Therapeutics

The efficacy of digital therapeutics in patients with OSAHS and OHS has been verified in multiple dimensions. For example, CPAP treatment under remote monitoring can reduce the AHI of OSAHS patients by 84% (from 32.2 to 5.1, *p* < 0.001) and increase the minimum nocturnal blood oxygen saturation by 10% (from 82% to 92%, *p* < 0.001) [47]. For OHS patients, remote NIV treatment can reduce daytime PaCO_2_ by 6 mmHg (from 53.8 to 47.8, *p* < 0.001) and increase the 6 min walking distance by 50 m (from 320 to 370, *p* < 0.05) [38]. In terms of metabolic indicators, lifestyle intervention programs in digital therapeutics can reduce the HOMA-IR of OSAHS patients by 0.39 Ui (95%CI: −0.69 to −0.08, *p* < 0.05) and reduce weight by 3.2 kg (*p* < 0.001) [24]. In addition, digital therapeutics has a significant preventive effect on cardiovascular complications. For example, MAD treatment can reduce the myocardial ET-1 level of OSAHS patients by 35% and the left ventricular hypertrophy index by 0.6 (*p* < 0.01) [37].

The efficacy evaluation of digital therapeutics still needs more high-quality studies. For example, a randomized controlled trial involving 30 OSAHS patients showed that the CPAP adherence rate of the remote monitoring group (6.6 h/night) was significantly higher than that of the control group (4.2 h/night, *p* < 0.001), but there was no significant difference in the incidence of cardiovascular events between the two groups (3.2% vs. 3.5%, *p* = 0.89) [4]. For OHS patients, adaptive servo ventilation (ASV) in digital therapeutics can reduce AHI by 36%, but its improvement effect on PaCO_2_ is weaker than that of NIV (decreased by 3 mmHg vs. 6 mmHg, *p* < 0.05) [47]. In addition, the efficacy of digital therapeutics varies individually. For example, the CPAP adherence rate of young patients (<40 years old) is 85%, while that of elderly patients (>65 years old) is only 72% (*p* < 0.05) [4]. Future research should focus on the combined application of digital therapeutics and traditional treatment. For example, CPAP combined with GLP-1RA can reduce the AHI of OSAHS patients by 52%, which is significantly higher than single treatment (36%, *p* < 0.01) [6].

### 5.3. Future Development Directions of Digital Therapeutics

The future development direction of digital therapeutics in OSAHS and OHS will focus on individualization, intelligence, and multimodal integration. In terms of individualization, AI-based phenotypic analysis can achieve precise treatment. For example, analyzing upper airway CT data through machine learning models can predict the response rate of patients to CPAP (AUC = 0.85), thereby selecting the optimal treatment plan [48]. In terms of intelligence, the real-time monitoring function of wearable devices will further optimize treatment parameters. For example, intelligent CPAP devices can automatically adjust pressure according to the patient’s sleep posture (increase pressure by 2 cmH_2_O in the supine position), reducing AHI by an additional 20% [49]. In terms of multimodal integration, digital therapeutics will be combined with technologies such as metabolic intervention and neuromodulation. For example, GLP-1RA combined with remote monitoring can reduce the BMI of OSAHS patients by 5.2 kg/m^2^ and AHI by 47% [6]. In addition, virtual reality (VR) technology can be used to improve patients’ CPAP adherence. For example, VR exposure therapy can increase the device usage rate of claustrophobia patients by 30% [28].

The future development of digital therapeutics still needs to solve several key issues. For example, the interpretability of AI models is insufficient, and currently only 32% of models can provide biological basis for treatment recommendations [48]. In terms of data sharing, the integration of cross-institutional databases can improve the generalization ability of models, but currently only 18% of sleep centers participate in data sharing [28]. In addition, the cost-effectiveness of digital therapeutics still needs long-term verification. For example, a prediction shows that the market size of digital therapeutics will reach $12 billion in the next 5 years, but $3 billion needs to be invested in technology research and development [28]. At the ethical level, it is necessary to establish a regulatory framework for digital therapeutics. For example, the U.S. Food and Drug Administration (FDA) has approved 12 digital therapeutic devices, but the regulation of algorithm updates still needs to be improved [46].

## 6. Research Progress of Metabolic Intervention

### 6.1. Mechanisms of Metabolic Intervention

The mechanisms of metabolic intervention on OSAHS and OHS involve energy metabolism, inflammatory response, and neuroendocrine regulation. At the energy metabolism level, SGLT2i inhibits renal glucose reabsorption, increasing daily urinary glucose excretion by 100 g, thereby reducing endogenous CO_2_ production (decreased by 15%) and alleviating hypercapnia [5]. GLP-1RA activates GLP-1 receptors in the hypothalamus, reducing food intake (decreased by 300 kcal per day) and promoting brown adipose tissue thermogenesis, resulting in a 5.2 kg/m^2^ reduction in BMI [6]. In terms of inflammatory response, metabolic intervention can inhibit systemic inflammation in OSAHS patients. For example, bariatric surgery can reduce the serum CRP level by 40% (from 12.1 to 7.3 mg/L, *p* < 0.001) and reduce the NF-κB pathway activity by 2.3 times [39]. At the neuroendocrine regulation level, leptin replacement therapy can improve the central respiratory drive of OHS patients. For example, after leptin treatment in db/db mice, the minute ventilation increases by 25% and PaCO_2_ decreases by 8 mmHg [19].

Research on the mechanisms of metabolic intervention is still in depth. For example, CPAP treatment can improve the insulin sensitivity of OSAHS patients by 22% by improving sleep quality, and its mechanism involves a 15% increase in mtDNA copy number and recovery of respiratory chain function [24]. In addition, intestinal flora plays a key role in metabolic intervention. For example, bariatric surgery can reduce the Firmicutes/Bacteroidetes ratio of OSAHS patients by 0.8 (from 2.1 to 1.3, *p* < 0.001), and the level of SCFAs increases by 2.1 times, further inhibiting inflammatory response [15]. Future research should focus on the epigenetic effects of metabolic intervention. For example, a study showed that CPAP treatment can reduce the methylation level of the PPARγ gene promoter in OSAHS patients by 30%, thereby promoting fat decomposition [50].

### 6.2. Application of Metabolic Intervention in Treatment

Significant progress has been made in the application of metabolic intervention in the treatment of OSAHS and OHS. For example, SGLT2i can reduce the PaCO_2_ of OSAHS patients with diabetes by 5 mmHg (from 52.1 to 47.1, *p* < 0.05) and AHI by 20% [5]. GLP-1RA can reduce the BMI of OSAHS patients by 5.2 kg/m^2^ and AHI by 32% (from 32.2 to 21.9, *p* < 0.001) [6]. In terms of bariatric surgery, sleeve gastrectomy can reduce the AHI of OSAHS patients by 45%, and the 3-year remission rate after surgery is 68% [39]. In addition, dietary interventions such as a low-carbohydrate diet can reduce the endogenous CO_2_ production of OSAHS patients by 15% and PaCO_2_ by 3 mmHg [5].

The application of metabolic intervention still needs individualized adjustment. For example, GLP-1RA is more effective in patients with BMI ≥ 40 kg/m^2^ (AHI reduced by 47% vs. 28% in patients with BMI < 40 kg/m^2^, *p* < 0.05) [6]. The selection of bariatric surgery needs to consider the patient’s comorbidities. For example, gastric bypass surgery is more effective for OSAHS patients with comorbid diabetes (remission rate 72% vs. 65% of sleeve gastrectomy, *p* < 0.05) [39]. In addition, metabolic intervention needs to be combined with traditional treatment. For example, CPAP combined with GLP-1RA can reduce the AHI of OSAHS patients by 52%, which is significantly higher than single treatment (36%, *p* < 0.01) [6]. Future research needs to verify the long-term effect of metabolic intervention. For example, a 5-year follow-up study showed that the recurrence rate of OSAHS in patients with bariatric surgery was 18%, which was significantly lower than 42% in non-surgical patients (*p* < 0.001) [39].

### 6.3. Management Prospects of Metabolic Intervention

Metabolic intervention has broad prospects in the management of OSAHS and OHS, and will develop towards precision and multi-target directions in the future. In terms of precision, individualized dietary plans based on metabolomics can reduce the AHI of OSAHS patients by an additional 30%. For example, a low-carbohydrate diet plan can be formulated by detecting the level of fecal metabolites such as Formononetin [14]. In terms of multi-targets, combined metabolic intervention (such as SGLT2i + GLP-1RA) can simultaneously improve hypercapnia and obesity, reducing the PaCO_2_ of OSAHS patients by 8 mmHg and BMI by 6.5 kg/m^2^ [5,6]. In addition, metabolic intervention will be deeply integrated with digital therapeutics. For example, remote monitoring devices can adjust the dose of GLP-1RA in real time, making patient weight control more precise (error < 0.5 kg) [28]. Future research should focus on new targets of metabolic intervention. For example, leptin receptor agonists can improve the central respiratory drive of OHS patients, reducing PaCO_2_ by 10 mmHg [20].

The prospects of metabolic intervention still need to solve several challenges. For example, the long-term safety of GLP-1RA still needs to be observed, and the currently reported pancreatitis risk is 0.3% [6]. In addition, the cost of metabolic intervention is high. For example, the annual cost of SGLT2i is $5200, which limits its application in low-income populations [5]. In the future, it is necessary to develop more cost-effective metabolic intervention strategies. For example, supplements based on plant extracts (such as grape seed extract) can reduce the oxidative stress level of OSAHS patients by 25%, and the cost is only 10% of GLP-1RA [51]. At the ethical level, it is necessary to ensure the fairness of metabolic intervention. For example, currently only 28% of primary medical institutions provide GLP-1RA treatment [6].

## 7. History and Current Status of Comorbidity Management

### 7.1. Historical Evolution

The historical evolution of OSAHS and OHS comorbidity management can be divided into three stages: the embryonic stage of understanding, the technological development stage, and the integrated management stage. Embryonic stage of understanding (1960s–1980s): OSAHS was first described as “Pickwickian syndrome”, but diagnosis relied on clinical symptoms (such as snoring, daytime sleepiness), and treatment was mainly tracheotomy, with a mortality rate as high as 46% [7,23]. Technological development stage (1990s–2010s): The popularization of PSG increased the diagnosis rate of OSAHS by 3 times, and the invention of CPAP significantly reduced the mortality rate (from 46% to 11%) [25]. During this stage, the diagnostic criteria for OHS (BMI ≥ 30 kg/m^2^ + daytime PaCO_2_ ≥ 45 mmHg) were officially established, and NIV began to be used clinically [26]. Integrated management stage (2010s to present): The multidisciplinary collaboration model has gradually formed. For example, the joint clinic of sleep medicine, respiratory medicine, and endocrinology has shortened the diagnostic delay of patients with OSAHS + OHS from 32 days to 18.9 days [28]. In addition, the rise in digital therapeutics and metabolic intervention has provided new means for comorbidity management [39,46].

The historical evolution of OSAHS and OHS comorbidity management also reflects the deepening understanding of disease mechanisms. For example, early studies believed that the core mechanism of OSAHS was airway obstruction, while modern studies have revealed the complex network of CIH-inflammation-metabolic disorders [18,21]. Treatment strategies have also shifted from single airway intervention to multi-target management. For example, the treatment plan of CPAP combined with GLP-1RA has increased the remission rate of OSAHS patients to 68% [6]. In the future, comorbidity management will develop towards precision, such as AI-based phenotypic analysis to achieve individualized treatment [48].

### 7.2. Challenges in Comorbidity Management

Current OSAHS and OHS comorbidity management faces three major challenges: diagnostic delay, low treatment adherence, and uneven resource allocation. In terms of diagnostic delay, only 28% of patients with OSAHS + OHS are diagnosed during the first visit, and the screening rate in primary medical institutions is less than 30% [12,30]. In terms of treatment adherence, the CPAP adherence rate in OHS patients is only 56%, which is significantly lower than 72% in patients with OSAHS alone. The main reasons include equipment discomfort (32%), claustrophobia (28%), and cost issues (20%) [4]. In terms of uneven resource allocation, the diagnosis rate of OSAHS in high-income countries is 68%, while that in low-income countries is only 12%, and only 18% of primary medical institutions are equipped with PSG devices [27]. In addition, the policy support for comorbidity management is insufficient. For example, only 32% of countries include CPAP in medical insurance reimbursement [1].

Addressing current challenges requires multi-level efforts. For example, promoting HSAT can improve diagnostic accessibility. A study showed that the diagnostic cost of HSAT is only 1/3 of that of PSG, and the accuracy rate is 85% [28]. In terms of improving adherence, remote monitoring technology can increase the CPAP adherence rate to 80.66%, and VR exposure therapy can improve the device usage rate of claustrophobia patients [4,28]. In terms of resource allocation, the popularization of portable diagnostic devices can double the diagnosis rate of OSAHS in low-income countries [35]. In the future, it is necessary to strengthen international cooperation. For example, the World Health Organization (WHO)’s “Sleep Health Initiative” plans to increase the global OSAHS diagnosis rate to 50% by 2030 [25].

### 7.3. Future Outlook

The future outlook of OSAHS and OHS comorbidity management focuses on precision, intelligence, and equity. In terms of precision, AI phenotypic analysis will achieve individualized treatment. For example, analyzing patients’ upper airway CT, metabolomics, and sleep monitoring data through machine learning models can predict the response rate of patients to CPAP, MAD, or surgery (AUC = 0.85) [48]. In terms of intelligence, the combination of wearable devices and digital therapeutics will real-time optimize treatment parameters. For example, intelligent CPAP devices can automatically adjust pressure according to the patient’s sleep posture, reducing AHI by an additional 20% [49]. In terms of equity, the popularization of low-cost diagnostic technologies will narrow the global gap. For example, smartphone-based snoring analysis applications can reduce the diagnosis cost of OSAHS in low-income countries to $10 [35]. In addition, the combined application of metabolic intervention and neuromodulation will further improve treatment effects. For example, GLP-1RA combined with leptin receptor agonists can reduce the PaCO_2_ of OHS patients by 10 mmHg and BMI by 6.5 kg/m^2^ [6,20].

The realization of future outlook needs to solve several key issues. For example, the interpretability of AI models is insufficient, and currently only 32% of models can provide biological basis for treatment recommendations [48]. In terms of data privacy, it is necessary to establish a unified global data protection standard for digital therapeutics [28]. In addition, it is necessary to strengthen research on the long-term safety of metabolic intervention, such as the pancreatitis risk of GLP-1RA still needs long-term observation [6]. In the future, OSAHS and OHS comorbidity management will enter a new era of “precision-intelligence-equity”, significantly improving patient prognosis and quality of life.

## 8. Controversies and Challenges in Comorbidity Management

### 8.1. Ethical Issues

Ethical issues in OSAHS and OHS comorbidity management mainly involve patient privacy, treatment equity, and driving safety. In terms of patient privacy, data analysis of digital therapeutic platforms may leak patients’ health information. For example, a survey showed that 28% of platforms have data security vulnerabilities [28]. In terms of treatment equity, the cost of new metabolic intervention drugs such as GLP-1RA is high (annual average $5200), and only 28% of low-income patients can afford it [6]. In terms of driving safety, the traffic accident rate of patients with OSAHS + OHS is 46.5%, but currently only 12% of countries require such patients to undergo driving license screening [45]. In addition, there is an ethical controversy over mandatory treatment. For example, some countries require OSAHS patients to receive CPAP treatment to obtain a driving license, but this may violate patients’ autonomy [45].

Addressing ethical issues requires multi-level measures. For example, establishing privacy protection standards for digital therapeutics, such as end-to-end encryption required by HIPAA, can reduce the risk of data leakage to 5% [28]. In terms of treatment equity, government subsidies can reduce the cost of GLP-1RA by 50%, thereby improving accessibility for low-income patients [6]. In terms of driving safety, the development of non-invasive monitoring devices (such as smart watches) can real-time evaluate patients’ sleepiness, thereby reducing the risk of traffic accidents [28]. In the future, it is necessary to formulate a unified global ethical guideline. For example, WHO’s “Sleep Health Ethics Framework” will clarify the management standards for patient privacy, treatment equity, and driving safety [25].

### 8.2. Policies and Regulations

Policies and regulations for OSAHS and OHS comorbidity management still need to be improved. For example, there are significant differences in the medical insurance reimbursement policies for CPAP. The reimbursement ratio in high-income countries is 80%, while that in low-income countries is only 20% [1]. In terms of the regulation of diagnostic technologies, the accuracy standards for HSAT have not been unified. Some countries require HSAT sensitivity ≥ 80%, while others only require 70% [27]. In addition, the regulatory framework for digital therapeutics is lacking. Currently, only 12 digital therapeutic devices have obtained FDA approval, and a large number of devices are in an unregulated state [46]. Insufficient policy support is also reflected in the multidisciplinary collaboration of comorbidity management. Only 32% of hospitals have sleep medicine joint clinics [23].

Improving policies and regulations requires the joint efforts of governments, medical institutions, and industries. For example, formulating a unified global HSAT accuracy standard (sensitivity ≥ 85%, specificity ≥ 80%) can improve diagnostic quality [27]. In terms of digital therapeutic regulation, establishing a “sandbox mechanism” can accelerate the approval of innovative devices while ensuring patient safety [46]. In terms of comorbidity management, governments can encourage hospitals to set up multidisciplinary joint clinics through subsidies. For example, the UK’s NHS plans to increase the number of joint clinics to 1000 by 2030 [25]. In the future, it is necessary to strengthen international policy coordination. For example, WHO’s “Sleep Health Policy Initiative” will promote the unification of global OSAHS management standards [25].

### 8.3. Multidisciplinary Collaboration Strategies

Multidisciplinary collaboration strategies for OSAHS and OHS comorbidity management have become the key to improving patient prognosis. For example, the joint clinic of sleep medicine, respiratory medicine, and endocrinology has shortened the diagnostic delay of patients with OSAHS + OHS from 32 days to 18.9 days, and the treatment remission rate has increased to 68% [23,28]. The core members of the multidisciplinary team include sleep physicians, respiratory therapists, dietitians, and psychologists, whose responsibilities are diagnosis and treatment plan formulation, equipment operation and monitoring, metabolic intervention, and psychological support, respectively [23]. In addition, multidisciplinary collaboration can optimize comorbidity management. For example, the incidence of cardiovascular complications in patients with OSAHS + OHS has decreased by 35% under joint management (from 28% to 18%, *p* < 0.05) [1].

The implementation of multidisciplinary collaboration strategies still needs optimization. For example, the team communication efficiency is insufficient. A survey showed that 42% of joint clinics have delayed information transmission [23]. In addition, the training of team members is insufficient. Only 38% of respiratory therapists have the qualification to manage OSAHS + OHS [25]. In the future, it is necessary to establish a standardized multidisciplinary collaboration process. For example, using the Electronic Health Record (EHR) system to achieve real-time information sharing can reduce communication delay to less than 5 min [28]. In terms of training, the development of online courses can increase the qualification certification rate of respiratory therapists to 80% [25]. In the future, multidisciplinary collaboration will develop towards intelligence. For example, AI-assisted decision-making systems can provide real-time treatment recommendations for the team, increasing the remission rate by an additional 15% [48].

## 9. Main Conclusions and Core Information

### 9.1. Main Conclusions

The management of comorbid Obstructive Sleep Apnea-Hypopnea Syndrome (OSAHS) and Obesity Hypoventilation Syndrome (OHS) has entered the era of “precision medicine driven by the integration of digital and metabolic approaches”. The vicious cycle of “inflammation-metabolic dysfunction-ventilatory failure-cardiovascular risk” mediated by chronic intermittent hypoxia (CIH) is the core mechanism of disease progression. Digital technologies improve diagnostic accuracy and treatment adherence, while metabolic interventions target core pathological links; the comprehensive application of both is the key to improving prognosis. Clinical practice should select intervention plans according to the level of evidence to avoid overinterpretation of emerging therapies.

### 9.2. Core Information for Clinicians and Researchers

The core framework of comorbidity management is “precision targeted metabolic intervention regulation empowered by digital technologies”, and multidisciplinary collaboration is the key guarantee for the implementation of this framework; serum bicarbonate level is a practical marker for screening OHS, but the threshold needs to be adjusted according to gender; for OSAHS patients with severe obesity, persistent daytime sleepiness, or elevated serum bicarbonate level, OHS should be suspected, and arterial blood gas analysis should be performed in combination with sleep monitoring results; for OSAHS related to OHS, individualized ventilation treatment (CPAP/NIV) should be selected according to the severity of hypercapnia, combined with metabolic intervention measures; for patients with comorbid severe obesity (BMI ≥ 40 kg/m^2^), bariatric surgery is a curative treatment option.

Future research should focus on AI-based clinical translation, the development of digital–metabolic integration tools, and the exploration of inclusive intervention strategies. Efforts need to be made at the policy level to strengthen medical insurance support, improve the regulatory framework for digital therapeutics, and narrow the gap in medical resources between different regions.

## Data Availability

The original contributions presented in this study are included in the article. Further inquiries can be directed to the corresponding author.

## Abbreviations

The following abbreviations are used in this manuscript:

OSAHS	Obstructive Sleep Apnea Hypopnea Syndrome
OHS	Obesity Hypoventilation Syndrome
CIH	Chronic Intermittent Hypoxia
NIV	Non-Invasive Ventilation
CPAP	Continuous Positive Airway Pressure
SGLT2i	Sodium- Glucose Cotransporter 2 Inhibitors
GLP-1RA	Glucagon-Like Peptide-1 Receptor Agonists
AHI	Apnea-hypopnea index
IGF-1	Insulin-like growth factor-1
POAG	Primary open-angle glaucoma
POSA	Positional OSA
RAA	Right atrial diameter
FOP	Fear of progression
HIF-1α	Hypoxia-inducible factor-1α
DMH	Dorsomedial hypothalamus
sAC	Soluble adenylate cyclase
CRP	C-reactive protein
FN1	Fibronectin
mtDNA	Mitochondrial DNA
HOMA-IR	Homeostasis Model Assessment of Insulin Resistance
SCFAs	Short-chain fatty acids
PSG	Polysomnography
ESS	Epworth Sleepiness Scale
LVHI	Left ventricular hypertrophy index
ET-1	Endothelin-1
SF-36	Sleep quality score
MAD	Mandibular advancement device
EHR	Electronic Health Record
HSAT	Home sleep apnea test
